# Intermittent fasting: consider the risks of disordered eating for your patient

**DOI:** 10.1186/s40842-023-00152-7

**Published:** 2023-10-21

**Authors:** Jack Blumberg, Samantha L. Hahn, Jesse Bakke

**Affiliations:** Central Michigan University College of Medicine, 1200 S. Franklin St, Mount Pleasant, MI 48859 USA

**Keywords:** Diabetes, Intermittent fasting, Health, Nutrition, Disordered eating, Eating disorders

## Abstract

This is a commentary on “Intermittent fasting: is there a role in the treatment of diabetes? A review of the literature and guide for primary care physicians” by Albosta et al. While this article adequately summarized the biochemical clinical advantages and limitations, we feel it failed to mention a few drawbacks, primarily the risk for disordered eating and eating disorders. Here we delve into the emerging data on intermittent fasting or time-restricted feeding in patient populations and urge clinicians to consider these risks prior to encouragement of intermittent fasting.

## Background

In this issue of BMC Clinical Diabetes and Endocrinology, Albosta et al. present a comprehensive review of the current literature as well as a preliminary guide for primary care physicians regarding the possible use of intermittent fasting for their patients. As documented in the review, intermittent fasting (or time restricted eating) may significantly improve a patient’s health in certain situations. The evidence suggests that intermittent fasting may improve cardiometabolic outcomes for patients with diabetes and pre-diabetes. However, while much work has been done to investigate the beneficial metabolic effects of intermittent fasting, there is a significant lack of insight into its potential drawbacks. This is a pivotal moment for the future of intermittent fasting and its role in clinical medicine in terms of achieving a higher level of precision when determining whether intermittent fasting may be helpful or harmful for a patients overall health. The crucial portion of this exploration is to understand in which scenario to deliver this therapy, and in which it may be prudent to avoid. An important risk that intermittent fasting may pose is the potential to increase risk of disordered eating, which has only recently started to be examined. The importance of eating disorders should not be understated. Eating disorders are marked by high morbidity and mortality, have a lifetime prevalence of 9%, and add significant burdening costs to the healthcare system [1].

## Intermittent fasting and risks of disordered eating and eating disorders

A cross-sectional Canadian study by Ganson et al. explored the association between intermittent fasting and disordered eating, among 2762 adolescent and young adults [2]. They surveyed participants on their use of intermittent fasting over the previous 12 months. In total, 47.7% of female participants, 38.4% of male participants, and 52.0% of transgender and gender non-conforming individuals reported having used intermittent fasting in the previous 12 months. These individuals also completed the Eating Disorder Examination Questionnaire. Women showed the most consistent associations between intermittent fasting and disordered eating behaviors, whereas men showed a significantly weaker correlation between the two. The results highlight the high prevalence of intermittent fasting across a national sample of Canadian teenagers and young adults and provide evidence for the higher likelihood of eating disorder behaviors and psychopathology among those who engage in intermittent fasting. This survey demonstrates that for adolescents and young adults, especially in women, the risk for developing disordered eating due to engaging in intermittent fasting is substantial.

In a similar study, Colombarolli et al*.* aimed to identify if a low-carb diet was related to disordered eating and if intermittent fasting was associated with further exacerbation of these symptoms [3]. The study comprised of 682 college students. Over one quarter (27%, n = 188) of respondents reported following a low-carb diet within the last three months. Thirty-one percent (n = 58) of these individuals reported combining intermittent fasting with a low-carb diet. Their results showed that a low-carb diet and intermittent fasting were both independently associated with higher likelihood of binge eating and food cravings compared to those who did not diet. Furthermore, individuals who engaged in both low-carb diets and intermittent fasting had substantially higher likelihood of binge eating and having food cravings than either intervention independently. Meaning that adding intermittent fasting to a low-carb diet was associated witho even more significant increases in disordered eating, especially binge eating. This study also exemplifies the negative food related cognitive effects intermittent fasting can produce such as food cravings. It is interesting to note that this study raises the possibility that intermittent fasting may hold a unique attribute that uniquely contributes to disordered eating, on top of the risks associated with traditional dieting.

A study designed to investigate the outcomes of a short intermittent fasting trial (28 days) was led by Langdon-Daly [4]. Unlike the previous studies, this trial included participants of all ages, not just young people, allowing for a better understanding of how intermittent fasting may affect individuals of all ages. The study involved initiating a 4 weeklong intermittent fasting period to compare the eating behavior of the participants before and after the fasting stage, while controlling for confounding variables. In healthy adults, adopting a 5:2 intermittent fasting pattern did not lead to an increase in disordered eating, including binge eating, and seemed to enhance all dietary, metabolic, and mood-related outcomes. Lower levels of disordered eating during the trial were linked with lower levels of baseline disordered eating, which introduces the possibility that intermittent fasting may be helpful for patients at low risk for disordered eating and harmful for those at high risk. Adjusting for baseline risk, this study found that undergoing a brief period of intermittent fasting did not lead to adverse effects as one could expect, but indeed had a positive reaction on them.

A meta-analysis by Donaldson, Freya ventured to compare the presence and differences in the effects of intermittent fasting and calorie restriction on eating disorders as well as general mood symptoms [5]. This analysis again was useful for assessing confounding variables due to the inclusivity of the population sample. In a particular controlled study within the analysis, a four-week period was used. During the four weeks of dieting, participants in both diet groups reported less binge-eating disorder symptoms, weight concerns, food cravings, and mood problems. After four weeks, the intermittent fasting group reported less anxiety about their appearance and weight than the calorie restriction group, as well as less concern about their eating habits. The authors concluded that while both approaches led to a significant reduction in disordered eating and mental health adversity, intermittent fasting was the clear winner, in agreement with other studies [4, 6]. However, these results should not automatically be extrapolated to a longer period because it is uncertain how easily these results can be maintained over time [7].

It is evident from the current literature that there are stark discrepancies in study findings across studies. The mixed findings could be due to a multitude of factors including differences in duration of intervention, population, or study design. First, with a shorter duration, weight loss may lead to a temporary increase in body satisfaction which would be reflected on shorter studies [8]. However, this weight loss is often difficult to maintain over longer periods of time which means when the weight does come back, body dissatisfaction will likely return as well. Second, there could be differences based on the study population. The data consistently showed that younger, more female populations were more likely to be negatively impacted by engaging in intermittent fasting versus middle aged populations. This point makes considering high-risk and low-risk groups crucial to understanding the data. Lastly, having multiple interventions or interventions with multiple parts (e.g., counseling, social support) could have obscured the data making it difficult to determine which intervention, or part of the intervention, is causing the effect. Further, individuals in the general population who are seeking out information on intermittent fasting are likely using online sources such as social media, which may be rife with misinformation that could be harmful, and those engaging in intermittent fasting also lack the clinical oversight that would be necessary to prevent or quickly identify disordered eating [9].

Currently, there is a reasonable consensus forming that intermittent fasting may be implemented in certain scenarios, namely for diabetes. The cruciality lies in the patient’s/physician’s ability to make an informed judgement on whether the prospective benefit is worth the risk for their patients. Narrowing in on disordered eating, the key seems to be using context to one’s advantage in this decision-making process. At this point in time based on existing data, the most reasonable clinical guide for a practitioner to use, in our opinion, is the following: Patients with an eating disorder or history of an eating disorder or disordered eating should never be encouraged to engage in intermittent fasting. Furthermore, extreme caution should be taken in recommending intermittent fasting to patients that carry risk factors for disordered eating such as being an adolescent/young adult, particularly those who identify as female or gender diverse populations.

This leads us to our belief that every time intermittent fasting is recommended to an individual from this demographic, an explicit warning is to be provided on the potential deleterious outcome of acquiring an eating disorder from this practice. As both Ganson et al*.* and *Schaumberg *et al*.* [10] highlighted in their research, this demographic with the help of social media introduces a massive patient population in which intermittent fasting has the potential to do more harm than good in the form of predisposing disordered eating, especially without any clinical indications. Patients receiving this guidance from their health care providers ought to and deserve to know this vital forewarning. Furthermore, we recommend that screening for disordered eating be routine in all clinical settings but that it be paramount to closely monitor for disordered eating in all patients for whom you recommend intermittent fasting. Patients with whom you believe intermittent fasting may be helpful, or if you suspect a patient has disordered eating, should be referred to a registered dietitian. Additionally, outside of disordered eating concerns, children, elderly, and pregnant/lactating women should not participate in intermittent fasting due to the lack of research on safety and efficacy in these populations.

## Conclusion

Intermittent fasting seems to be an effective way to treat certain medical conditions but should only be prescribed by knowledgeable healthcare providers using thoughtful discretion and with substantial disclaimers. Furthermore, it is wise to recommend providers in the field seek training for disordered eating as well as be able to skillfully identify warning signs using proper screening tools. It would benefit their practice to test for eating disorders in all patients, but especially those to whom they are giving serious consideration in making a recommendation of intermittent fasting, because disordered eating and eating disorders are frequently misdiagnosed and underdiagnosed. In this way, they can be proactive instead of reactive to connect them with a specialist, such as a registered dietitian, psychologists, and psychiatrist. Although this is a great start in approaching the challenges that intermittent fasting presents in its utility in medicine, there is still a large amount of work to be done. With more clinical data collected in the future, we will resolve existent uncertainty and shape accurate parameters in which physicians may safely instrument intermittent fasting.

Indeed however, it would be inappropriate to refrain from mentioning the effect that regularly scheduled eating, which is comprised of three full meals plus two snacks per day, has on disordered eating. In fact, Dalle Grave, Riccardo et al*.* demonstrated in their work that regular eating in conjunction with evidence-based enhanced cognitive behavior therapy (CBT-E) resulted in a quick reduction in the frequency of binge-episodes in individuals with binge-eating disorder and bulimia nervosa [11]. Conversely, intermittent fasting can lead to restrict-then-binge cycles, which would further exacerbate symptoms among those with eating disorders. Another alternative paradigm compared to intermittent fasting that is garnering more attention and worth exploring is intuitive eating. Intuitive eating has the benefit of being successful at improving both disordered eating [12] and physical outcomes but is more sustainable long-term and does not incur the potential risks associated with intermittent fasting [13]. As always, the benefits should be carefully weighed against the risks in deliberating the most suitable treatment plan for the patient.

## Data Availability

Data sharing not applicable to this article as no datasets were generated or analyzed during the current study.
